# Survival prediction of patients with sepsis from age, sex, and septic episode number alone

**DOI:** 10.1038/s41598-020-73558-3

**Published:** 2020-10-13

**Authors:** Davide Chicco, Giuseppe Jurman

**Affiliations:** 1 Krembil Research Institute, Toronto, ON Canada; 2 Fondazione Bruno Kessler, Trento, Italy

**Keywords:** Infection, Risk factors, Outcomes research, Biomedical engineering, Information technology, Computer science, Computational science

## Abstract

Sepsis is a life-threatening condition caused by an exaggerated reaction of the body to an infection, that leads to organ failure or even death. Since sepsis can kill a patient even in just one hour, survival prediction is an urgent priority among the medical community: even if laboratory tests and hospital analyses can provide insightful information about the patient, in fact, they might not come in time to allow medical doctors to recognize an immediate death risk and treat it properly. In this context, machine learning can be useful to predict survival of patients within minutes, especially when applied to few medical features easily retrievable. In this study, we show that it is possible to achieve this goal by applying computational intelligence algorithms to three features of patients with sepsis, recorded at hospital admission: sex, age, and septic episode number. We applied several data mining methods to a cohort of 110,204 admissions of patients, and obtained high prediction scores both on this complete dataset (top precision-recall area under the curve PR AUC = 0.966) and on its subset related to the recent Sepsis-3 definition (top PR AUC = 0.860). Additionally, we tested our models on an external validation cohort of 137 patients, and achieved good results in this case too (top PR AUC = 0.863), confirming the generalizability of our approach. Our results can have a huge impact on clinical settings, allowing physicians to forecast the survival of patients by sex, age, and septic episode number alone.

## Introduction

Sepsis is a dangerous condition triggered by an immune overreaction to an infection. According to the World Health Organization (WHO) estimates, sepsis affects more than 30 million people yearly worldwide , causing approximately 6 million deaths^1^, and causing more than US$24 billion healthcare related costs annually just in United States^2^. The scientific community is still investigating sepsis etiology^3^, whilst its management^4–6^ is troublesome due to the high disease’s complexity and heterogeneity^7,8^. A further complexity factor lies in a more restrictive definition of sepsis introduced in 2016^9^; the new definition, named Sepsis-3^10^, now requires the presence of additional organ dysfunctions for the condition to be labelled as sepsis. Although the usefulness of Sepsis-3 has recently been validated^11^, it is still debated within the medical community^12^. Additionally, early detection is critical to managing the attack and obtaining a favorable outcome, as Sepsis can kill a patient in as little as an hour.

### Prediction of survival of patients with sepsis

Medical literature is rich of general purpose articles on sepsis^13^, and quest for biomarkers in clinical settings have now spanned several decades, with papers dating back to early seventies still relevant today^14^. Initially, the core of the researches focused on clinical trials aimed at identifying therapeutic factors representing potential targets for novel or repurposed drugs. The crucial change of pace occurred in the early 2000s, when broad epidemiological data begun being publicly available, yielding the appearance of large retrospective studies^2,15^. Indeed, such recent influx of data has resulted in a steady flow of medical and computer science studies in which researchers have used various data science techniques to find associations between clinical factors and sepsis outcomes, with patient survival being among the most important. Contributing to the landscape, the practitioners’ community started introducing different early warning scores, such as physiological monitoring systems for detecting of acutely deteriorating patients^16^. A small group of scores quickly gained popularity in the clinical settings, thus becoming *de facto* standards for benchmarking studies: APACHE^17^, SAPS^18^, SOFA^19^ and qSOFA score^10^. Adding to such established community shared scorxes, different formulas have been recently defined in the literature involving alternative variables: for instance, the dynamic pulse pressure and vasopressor (DPV), the delta pulse pressure ($\Delta$PP)^20^ and the sepsis hospital mortality score (SHMS)^21^. However, although early warning scores have been widely adopted, there is only limited evidence of their effectiveness in the improvement of patient outcome^16^. Among all statistical methods, algorithms based on multivariate (Cox) regression on clinical variables have played a key role^22,23^, since back in the early years^24^ to nowadays^25^. Notably, the features involved in these methods are not limited to clinical variables: in the last few years a number of teams tried alternative elements from modern omics technologies, such as metabolomics^26^, SNPs genomics^27^, circulating microRNA^28^, blood metabolites^29^ or lymphocytes apoptosis^30^, often coupled with more classical biomarkers and compared with the different scores. Unfortunately, these statistical based approaches proved to be rather limited in their performances, with only a tiny fraction of studies achieving acceptable level of efficacy^31^. Indeed, Gwadry-Sridhar and colleagues^32^ claimed superiority of decision trees over regression methods already in 2010.

### Sepsis and machine learning

More recently machine learning has become the major player in the predictive analysis of sepsis data, leading to a massive wave of studies targeting different aspects of the problem, from the general issue^33–51^, to more specific objectives or methods. For instance, many studies have been defining, combining and validating score risks^52,53^, predicting early onset^54–56^, or focusing on pediatric aspects^57^ or on the immediate applicability to clinical practice^58–60^. Longitudinal studies have also appeared^61–63^, together with methods integrating alternative data sources such as omics^64^ and others. In the end of the 2010s, the computational intelligence revolution entered the playground too, and deep learning approaches flooded the specialized journals^65–73^, also considering the interpretability issue^74,75^. Fleuren et al.^76^ published comprehensive review of the different aspects. As mentioned earlier, many of these studies have been possible thanks to the public availability of curated clinical datasets related to sepsis. Among these datasets, we point out the Surviving Sepsis Campaign initiative^77,78^ (albeit not fully publicly released), the Medical Information Mart for Intensive Care database (MIMIC-III)^79^ and the electronic Intensive Care Unit (eICU) Collaborative Research Database^80^, which stand our for their completeness and integrity. Additionally, it is worth mentioning some notable studies aimed at identifying a restricted number of sepsis survival predicting features^81,82^: for instance, six predictors by Mao and colleagues^83,84^, five main predictive features by Shukeri et al.^85^, and three blood biomarkers by Dolin and coauthors^86^.

### This study

In the present manuscript, we take a similar approach: our driving goal is the prediction of the binary survival in a large cohort of Norwegian patients originally introduced and made public by Knoop and colleagues^87^. In addition to this prognostic task, as a distinguishing feature we also aim at proving that a minimal set of predictors can adequately predict the survival status. To further confirm the validity of our approach, we show that our approach can also be applied to an external South Korean dataset having the same clinical features, that we use as validation cohort. As a major outcome of such quest and improving over the published literature, we discovered that a single clinical factor, namely the progressive hospitalization episode, coupled with the two basic personal elements age and sex, can effectively predict the survival of the patients. Notably, we carried out the analysis both on the whole cohort, originally called *primary cohort*, corresponding to the admissions of the patients affected by sepsis potential preconditions (ante Sepsis-3 definition), and on a subset of the data including only the patients’ admissions defined by the novel Sepsis-3 definition, called *study cohort*. We then repeated the same analysis entirely on the validation cohort, and finally trained our models on the primary and study cohorts to apply them afterwards to the validation cohort. For the first time, we show that is possible to apply machine learning to sex, age, and septic episode number collected from admission clinical records to predict the survival of the patients who had sepsis. Our very small set of detected predictors represent a sensible compromise between accuracy and simplicity of the model, requiring few resources as collected data. This balance is critical when considering the translation to clinical practice, which especially for sepsis management is rarely successful^58^ and not easily integrated with clinicians’ activities^88^. Although a number of digital handling proposals have appeared in the literature^89^, the impact of sepsis of Food and Drugs Administration (FDA) approved Software as Medical Devices (SaMD)^90^, for example, is yet far from being widespread, with perhaps the Sepsis Prediction and Optimization of Therapy system (SPOT)^91^ as the most famous example. This given, having a simple albeit accurate predictive test on patient survival as presented here is a promising initial step towards the development of a machine learning-based tool supporting clinicians in everyday practice.

We organized the rest of the manuscript as follows. After this Introduction, we describe the dataset analyzed (Datasets), and the results we obtained (Results) Afterwards, we discuss the impact and consequences of our results, and limitations and future developments of study (Discussion), and describe the methods we employed ((Methods).

## Datasets

### Primary cohort and study cohort

We analyzed a dataset made of 110,204 admissions of 84,811 hospitalized subjects between 2011 and 2012 in Norway who were diagnosed with infections, systemic inflammatory response syndrome (SIRS), sepsis by causative microbes, or septic shock^87^. The data come from the Norwegian Patient Registry^92^ and the Statistics Norway agency^93^.

For each patient admission, the dataset contains sex, age, septic episode number, hospitalization outcome (survival), length of stay (LOS) in the hospital, and one or more codes of the International Classification of Diseases 10th revision (ICD-10) describing the patient’s disease (Table 1). Since the main goal of this study is to predict the survival of the patient, we discarded the length of stay because it strongly relates to the likelihood to survive: the longer the patient has to stay in the hospital, the less likely she/he will survive. The survival variable relates to the hospital length-of-stay, which ranges in the [0, 499] days interval and has mean of 9.351 days. Our prediction therefore refers to the likelihood of a patient to survive or decease in the 9.351 days after the collection of her/his medical record, in the hospital.

The admissions are of 57,973 of men and of 52,231 of women, ranging from 0 to 100 years of age (Table 2 and Table 3). Most of the admissions (76.96%) relate to the first septic episode. More information about the dataset can be found in the original study^87^.

The original dataset curators Knoop et al.^87^ called the complete dataset with 110,204 admissions the *primary cohort*. From the primary cohort, they selected the admissions respecting the *Sepsis-3* definition of sepsis (at least one several infection or sepsis related ICD-10 code and at least one codes for acute organ dysfunction)^9^, and they called this subset the *study cohort*.

Since the data of the primary cohort were recorded before the Sepsis-3 definition emerged in 2016, we cannot know if a patient diagnosed with an ICD-10 code related to sepsis actually had an organ dysfunction afterwards. Therefore, we cannot know if these admissions can be considered related to sepsis by the current Sepsis-3 definition today. What we know, instead, is that the conditions of the primary cohort (infections, systemic inflammatory response syndrome (SIRS), sepsis by causative microbes, or septic shock) might have lead to sepsis. To reflect this information, we call these conditions *sepsis potential preconditions* in this study. We decided to consider both the primary cohort and the study cohort for our analysis because consensus on a unified definition of sepsis has not been reached by the medical community yet^94^.

We took the original dataset^95^ and applied the same selection, generating a study cohort having a different size from the one of Knoop et al.^87^: while their study cohort contained 18,460 admissions, our study cohort contains 19,051. We were unable to obtain the original study cohort subset from Knoop unfortunately.

Both the primary cohort and our study cohort are positively imbalanced (Table 2). The primary cohort contains 102,099 admissions of patients who survived (92.65% positives) and 8,105 admissions of patients who deceased (7.35% negatives). Our study cohort contains 15,445 admissions of patients who survived (81.07% positives) and 3,606 admissions of patients who deceased (18.93% negatives).

**Table 1:** Meanings, measurement units, and intervals of each feature of the dataset.

Feature	Explanation	Measurement	Range
Age	Age of the patient at the hospital stay	Years	[0, ..., 100]
Episode number	Number of septic episodes experienced by the patient	Integer	[1, ..., 5]
Sex	0: male; 1: female	Binary	0, 1
[Target] survival	0: dead; 1: alive	Boolean	0, 1

Ranges refer both to the primary cohort and the study cohort. We used survival as prediction the target in this manuscript.

**Table 2:** Statistical quantitative description of the category features.

Category feature	Primary cohort		Study cohort	
	#	%	#	%
Survival (0: dead)	8105	7.35	3606	18.93
Survival (1: alive)	102,099	92.65	15,445	81.07
Sex (0: male)	57,973	52.61	10,505	55.14
Sex (1: female)	52,231	47.39	8546	44.86

#: Number of admissions. %: percentage of admissions. Primary cohort full sample: 110,204 admissions. Study cohort full sample: 19,051 admissions.

**Table 3:** Statistical quantitative description of the numeric features.

Numeric feature	Primary cohort			Study cohort		
	Median	Mean	$\sigma$	Median	Mean	$\sigma$
Age	68	62.74	24.13	77	72.50	18.61
Episode number	1	1.35	0.75	1	1.40	0.75

Primary cohort full sample: 110,204 admissions. Study cohort full sample: 19,051 admissions. $\sigma$: standard deviation.

**Figure 1:**
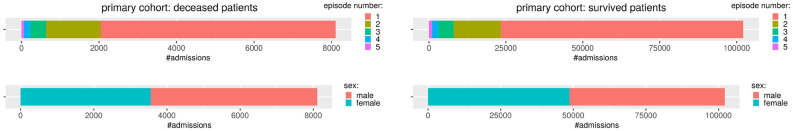
Primary cohort: stacked barplots of the distribution of categories. Distribution of sepsis episode number and sex of the admissions of patients who deceased (left) and survived (right). Admissions of survived patients: positives data instances (class 1). Admissions of deceased patients: negative data instances (class 0).

**Figure 2:**
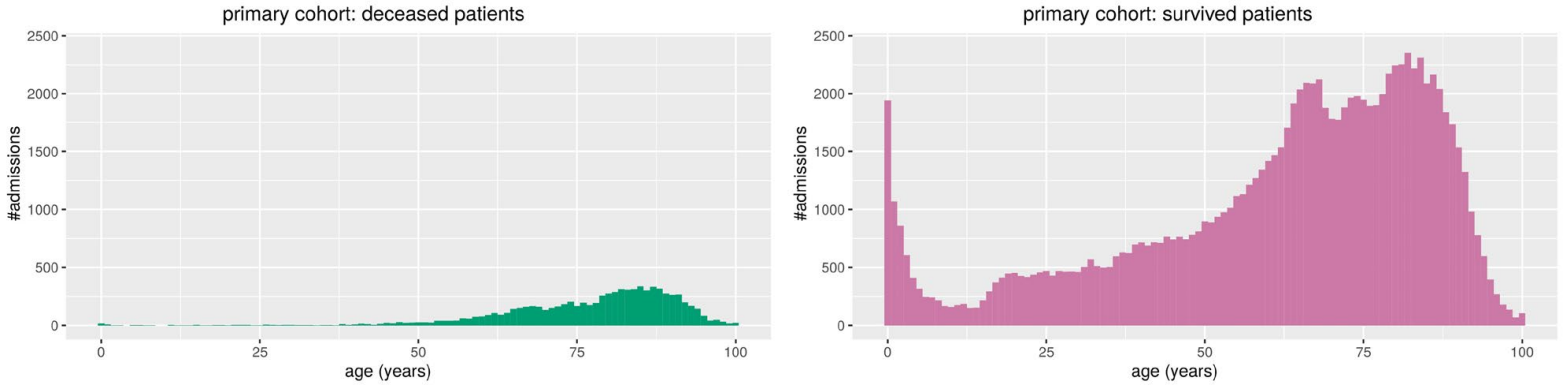
Primary cohort: histograms of the patients’ ages in relation with the number of admissions. On the left, the admissions of the patients who deceased. On the right, the admissions of patients who survived. Admissions of survived patients: positives data instances (class 1). Admissions of deceased patients: negative data instances (class 0).

**Figure 3:**
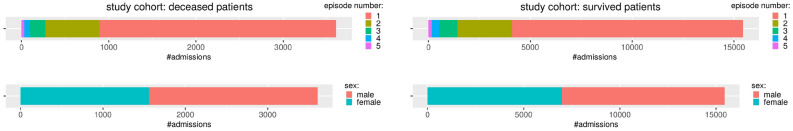
Study cohort: stacked barplots of the distribution of categories. Distribution of sepsis episode number and sex of the admissions of patients who deceased (left) and survived (right). Admissions of survived patients: positives data instances (class 1). Admissions of deceased patients: negative data instances (class 0).

**Figure 4:**
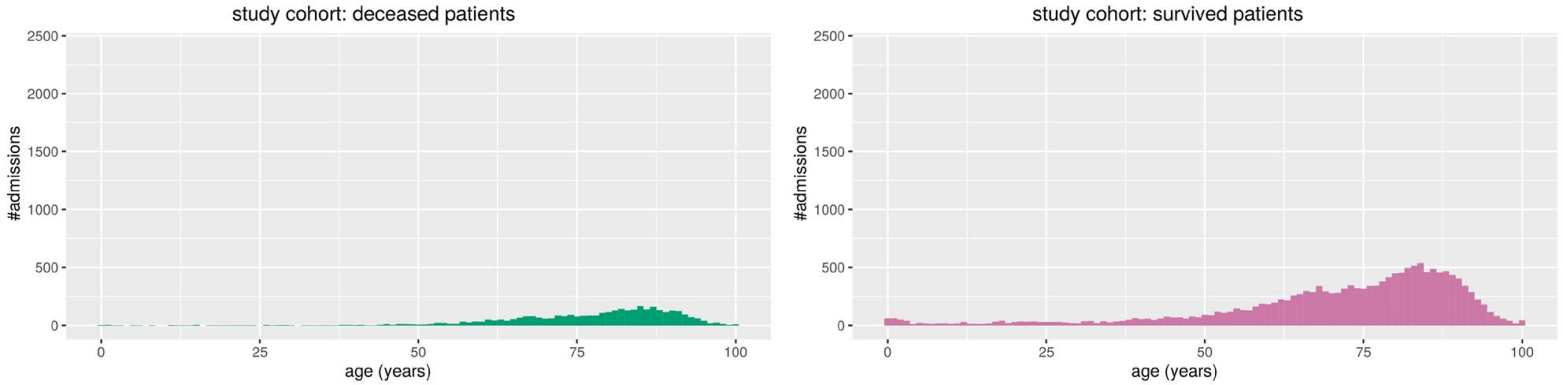
Study cohort: histograms of the patients’ ages in relation with the number of admissions. On the left, the admissions of the patients who deceased. On the right, the admissions of patients who survived. Admissions of survived patients: positives data instances (class 1). Admissions of deceased patients: negative data instances (class 0).

We report the stacked barplots of sex and disease episode number (Fig. 1) and the histogram of the age distribution (Fig. 2) for the primary cohort; we report the stacked barplots of sex and sepsis episode number (Fig. 3) and the histogram of the age distribution (Fig. 4) for the study cohort.

### Validation cohort

To confirm our findings, we also applied our methods to a dataset of South Korean critically ill patients whose medical records were collected between between January 2007 and December 2015 and publically released by Lee and colleagues^96^. From their original dataset, we selected the data of 137 patients who had already 1 or 2 septic episodes.

Since all these data were recorded before 2016, they are associated to a definition of sepsis earlier than *Sepsis-3*.

The dataset already contained the sex and age features, while we deduced the septic episode feature by selecting all the patients that already had a sepsis before the surgery (“Preop shock = 1”), and devided them between the ones that had a second sepsis afterwards (“new sepsis = 1”) and the ones that did not have it (“new sepsis = 0”).

The 137 patients of our validation cohort are 59.54 years old on average (median: 60), 47 women and 90 men. Among them, 115 had one septic episode and 22 had two septic episodes, while 113 survived and 24 deceased. Regarding the dataset imbalance, this validation cohort is positively imbalanced, having 82.482% positive data instances and 17.518% negative data instances.

More information about this dataset can be found in the original study^96^.

## Results

In this section we first describe the results we obtained through the traditional univariate biostatistics tests (Statistical correlations) and then the results we achieved through the machine learning classifiers on the primary and study cohorts (Survival predictions), and on the external validation cohort (Validation on external cohort).

### Statistical correlations

We applied some traditional biostatistics tests (Biostatistics univariate tests) to evaluate univariate associations between all three feature variables and survival status on the primary cohort. Their results showed they were statistically significant with $p<0.001$ (Table 4).

**Table 4:** Results of the application of univariate biostatistics tests between each feature and the survival target, in the primary cohort.

Feature	Mann–Whitney	Chi-squared
	Test *p* value	Test *p* value
Age	$< 2.20 \times 10^{-16}$	
Episode number	$1.72 \times 10^{-05}$	
Sex		$5 \times 10^{-04}$

Mann–Whitney test *p*-value: probability value generated by the application of the Mann–Whitney *U* test to the corresponding feature and survival. chi-squared test *p*-value: probability value generated by the application of the chi-squared test to sex and survival. We reported the features in alphabetical order.

The results of these tests state there are statistically meaningful relationships between age and survival, between disease episode number and survival, and between sex and survival. These results confirm that we can use these three clinical factors as predictive features to forecast survival.

### Survival predictions

We report the results of our machine learning predictions made on the primary cohort and on the validation cohort, measured with traditional confusion matrix rates, in Table 5. As mentioned earlier, we consider positive data instances the admissions of the survived patients (class 1), and negative data instances the admissions of the deceased patients (class 0).

**Table 5:** Results of the survival prediction made with machine learning classifiers, with training phase and testing phase done on the Norwegian primary cohort or study cohort^87^.

Method	PR AUC	ROC AUC	TP rate	TN rate	PPV	NPV	MCC	$\hbox {F}_1$ score	accuracy
**Training and testing on the primary cohort**									
Radial SVM	*0*.966*	0.701*	0.492	0.807	0.970*	0.112	+ 0.157*	0.652	0.515
Gradient boosting	*0*.966*	0.690	0.905*	0.179	0.934	0.126	+ 0.061	0.916*	0.851*
Naïve Bayes	0.954	0.649	0.553	0.745	0.965	0.117	+ 0.156	0.703	0.567
Linear regression	0.941	0.599	0.836	0.361	0.943	0.149	+ 0.135	0.886	0.801
Linear SVM	0.860	0.586	0.205	0.898*	0.896	0.210*	+ 0.104	0.333	0.337
**Training and testing on the study cohort**									
Linear SVM	*0*.860*	0.586*	0.205	0.898*	0.896*	0.210	+ 0.104*	0.333	0.337
Radial SVM	0.858	0.586*	0.408	0.718	0.861	0.222	+ 0.102	0.553	0.467
Gradient boosting	0.856	0.574	0.837*	0.208	0.822	0.231	+ 0.038	0.819*	0.718*
Naïve Bayes	0.841	0.562	0.405	0.718	0.861	0.220	+ 0.100	0.551	0.465
Linear regression	0.826	0.541	0.764	0.318	0.828	0.239*	+ 0.074	0.794	0.679

Mean results of 100 executions with random selection of the elements in the training set and test set, with ROSE oversampling^97^ applied to the training set. Admissions of survived patients: positives data instances (class 1). Admissions of deceased patients: negative data instances (class 0). Linear SVM: support vector machine with linear kernel. Optimized cost regularization hyper-parameter of the linear SVM, most frequently selected C by the MCC-based grid search: $C=0.01$ for primary cohort (63 times out of 100) and $C=0.001$ for study cohort (51 times out of 100). Radial SVM: support vector machine with radial Gaussian kernel. Optimized cost regularization of the radial SVM, most frequently selected C by the MCC-based grid search: $C=0.1$ for the primary cohort (56 times out of 100) and for the study cohort (51 times out of 100). MCC: Matthews correlation coefficient. MCC worst value $= -\;1$ and best value $= +\;1$. TP rate: true positive rate, sensitivity, recall. TN rate: true negative rate, specificity. PR: precision-recall curve. PPV: positive predictive value, precision. NPV: negative predictive value. ROC: receiver operating characteristic curve. AUC: area under the curve. $\hbox {F}_1$ score, accuracy, TP rate, TN rate, PPV, NPV, PR AUC, ROC AUC: worst value $= 0$ and best value $= +\;1$. We report the formulas of these rates in the Supplementary Information. ROSE minority class probability: $p=0.5$ for SVMs; $p=0.38$ for gradient boosting, naïve Bayes, and linear regression in the primary cohort; $p=0.45$ for gradient boosting, naïve Bayes, and linear regression in the study cohort. We highlighted in italic and with an asterisk * the top result for each statistical indicator. We report the mean scores with the standard deviations in Supplementary Table S1.

We report two scores considering all the possible confusion matrix thresholds (precision-recall curve and receiver operating characteristic curve), and seven scores computed by artificially setting the confusion matrix threshold to 0.5 (TP rate, TN rate, PPV, NPV, MCC, $\hbox {F}_1$ score, and accuracy). Since the main goal of our study is to predict the survived patients (positive data instances), and the inclusion of all the possible confusion matrix thresholds is more informative than the usage of an heuristic cut-off, we focused on the precision-recall area under the curve (PR AUC) as principal indicator (Table 5).

In the primary cohort, which contained admissions of patients diagnosed with sepsis before Sepsis-3, radial SVM and gradient boosting outperformed the other methods by achieving PR AUC = 0.966 and ROC AUC close to 0.7. Gradient boosting resulted being very efficient when predicting the survived patients, by achieving TP rate = 0.905, followed linear regression, that reached sensitivity = 0.805. Regarding the identification of deceased patients, the two SVM models attained the top TN rates: 0.898 for the linear SVM and 0.807 for the radial SVM.

All the five models obtained very high positive predictive values (PPVs), from linear SVM achieving 0.896 to radial SVM reaching the almost perfect value of 0.970. All the five methods, also, had low negative predictive values (NPVs), ranging from 0.112 to 0.210, which resulted in Matthews correlation coefficients, too. Regarding $\hbox {F}_1$ score and accuracy, four methods obtained high or very high results, with top performance reached by gradient boosting ($\hbox {F}_1$ score = 0.916 and accuracy = 0.851), while linear SVM achieved low scores on both these rates (Table 5).

In the study cohort, which contains admissions of patients diagnosed with sepsis based on the 2016 Sepsis-3 definition, the results were similar to those seen in the primary cohort, albeit a little lower.  (Table 5). All the five models obtained very high PR AUC, with linear SVM obtaining the top score of 0.860. Regarding the ROC AUCs, the two support vector machines gained the best results with 0.568 both. Gradient boosting and linear regression were capable to correctly predict most of the survived patients, reaching sensitivity scores of 0.837 and 0.764, respectively. And linear SVM was the best at predicting deceased patients, with a specificity score of 0.898.

Regarding precision, all the five methods were capable to make accurate positive predictions, with linear SVM obtaining again the best PPV (0.896). Similar to the primary cohort, they also all had low NPV values (ranging from 0.210 to 0.239), which was reflected in their Matthews correlation coefficients. The low results on the NPVs are reflected in the Matthews correlation coefficients, too. Gradient boosting gained high values for $\hbox {F}_1$ score and accuracy also in the primary cohort (0.819 and 0.718, respectively), followed by linear regression (Table S1).

### Validation on external cohort

To further verify the predictive power and the generalizability of our classifiers, we performed two additional analyses involving an external validation cohort containing medical records of patients from South Korea (Datasets)^96^.

In the first analysis, we both trained and tested our models on this external validation cohort, and reported the results (Table 6). In the second analysis, we trained our models on the Norwegian primary cohort or study cohort, applied the trained models to the external validation cohort, and reported the results (Table 7).

**Table 6:** Results of the survival prediction made with machine learning classifiers on the South Korean external validation cohort^96^.

Training and testing on the validation cohort									
Method	PR AUC	ROC AUC	TP rate	TN rate	PPV	NPV	MCC	$\hbox {F}_1$ score	accuracy
Linear SVM	*0*.899*	0.676	0.911	0.388	0.873	0.490	+ 0.309	0.889	0.818
Naïve Bayes	0.887	0.713*	0.899	0.527*	0.891*	0.538	+ 0.417*	0.893	0.828*
Gradient boosting	0.883	0.682	0.912	0.448	0.885	0.540*	+ 0.378	0.895*	0.828*
Linear regression	0.880	0.689	0.849	0.530	0.885	0.458	+ 0.350	0.863	0.788
Radial SVM	0.873	0.642	0.929*	0.226	0.849	0.465	+ 0.179	0.883	0.806

Mean results of 100 executions with random selection of the elements in the training set and test set, with ROSE oversampling^97^ applied to the training set. In this analysis, both the training phase and the testing phase happened on the validation cohort. $\sigma$: standard deviation. Admissions of survived patients: positives data instances (class 1). Admissions of deceased patients: negative data instances (class 0). Linear SVM: support vector machine with linear kernel. Optimized cost regularization hyper-parameter of the linear SVM, most frequently selected C by the MCC-based grid search: $C=0.1$ (59 times out of 100). Radial SVM: support vector machine with radial Gaussian kernel. Optimized cost regularization of the radial SVM, most frequently selected C by the MCC-based grid search: $C=0.1$ (70 times out of 100). MCC: Matthews correlation coefficient. MCC worst value $= -\;1$ and best value $= +\;1$. TP rate: true positive rate, sensitivity, recall. TN rate: true negative rate, specificity. PR: precision-recall curve. PPV: positive predictive value, precision. NPV: negative predictive value. ROC: receiver operating characteristic curve. AUC: area under the curve. $\hbox {F}_1$ score, accuracy, TP rate, TN rate, PPV, NPV, PR AUC, ROC AUC: worst value $= 0$ and best value $= +\;1$. ROSE *p*-value: 0.5 for all. We report the results with standard deviations in  Table S2 and the formulas of the statistical indicators in the Supplementary Information. We highlighted in italic and with an asterisk * the top result for each statistical indicator.

#### Train and test on the external validation cohort

Our results we report show that all our five methods (naïve Bayes, linear SVM, radial SVM, gradient boosting, and linear regression) are capable of efficiently predicting survival not only when trained and tested on the Norwegian cohorts, but also when trained and tested on another external dataset (Table 6). These results confirm the generalizability of our approach.

All the classifiers, in fact, obtained high PR AUC ranging from 0.873 (radial SVM) to 0.899 (linear SVM), and were able to correctly classify most of the positive data instances (minimum TP rate = 0.849) and most of the positive predictions (minimum PPV = 0.849). Only naïve Bayes and linear regression were able to correctly classify most of the negative data instances and correctly make most of negative predictions (specificity and NPV greater than 0.5 for both the methods).

Also the other indicators show good scores (ROC AUC and MCC) or optimal scores (accuracy and $\hbox {F}_1$ score for all the five classifiers, Table 6).

**Table 7:** Results of the survival prediction made with machine learning classifiers, including standard deviation, with training phase done on the Norwegian primary cohort or study cohort^87^ and testing phase done on the South Korean external validation cohort^96^.

method	PR AUC	ROC AUC	TP rate	TN rate	PPV	NPV	MCC	$\hbox {F}_1$ score	accuracy
**Train on primary cohort and test on validation cohort**									
Naïve Bayes	0.848*	0.565*	0.715	0.415*	0.852*	0.236*	+ 0.107*	0.777	0.663
Gradient boosting	0.843	0.527	0.953	0.035	0.823	0.123	– 0.018	0.882	0.792*
Radial SVM	0.821	0.514	0.949	0.013	0.819	0.040	– 0.068	0.879	0.785
**Train on study cohort and test on validation cohort**									
Gradient boosting	0.863*	0.552	0.973*	0.061	0.830	0.739*	+ 0.130*	0.895*	0.814*
Naïve Bayes	0.848	0.566*	0.747	0.386*	0.851*	0.244	+ 0.113	0.795	0.683
Radial SVM	0.829	0.537	0.955	0.011	0.820	0.043	– 0.068	0.882	0.789
Linear regression	0.824	0.499	0.956	0.042	0.824	0.166	– 0.005	0.885	0.796

Mean results of 100 executions with random selection of the elements in the training set and test set, with ROSE oversampling^97^ applied to the training set. $\sigma$: standard deviation. Admissions of survived patients: positives data instances (class 1). Admissions of deceased patients: negative data instances (class 0). Linear SVM: support vector machine with linear kernel. Optimized cost regularization hyper-parameter of the linear SVM, most frequently selected C by the MCC-based grid search: $C=0.01$ for primary cohort (63 times out of 100) and $C=0.001$ for study cohort (51 times out of 100). Radial SVM: support vector machine with radial Gaussian kernel. Optimized cost regularization of the radial SVM, most frequently selected C by the MCC-based grid search: $C=0.1$ for the primary cohort (56 times out of 100) and for the study cohort (51 times out of 100). MCC: Matthews correlation coefficient. MCC worst value $= -\;1$ and best value $= +\;1$. TP rate: true positive rate, sensitivity, recall. TN rate: true negative rate, specificity. PR: precision-recall curve. PPV: positive predictive value, precision. NPV: negative predictive value. ROC: receiver operating characteristic curve. AUC: area under the curve. $\hbox {F}_1$ score, accuracy, TP rate, TN rate, PPV, NPV, PR AUC, ROC AUC: worst value $= 0$ and best value $= +\;1$. We report the formulas of these rates in the Supplementary Information, and the same results including the standard deviations in Table S3. ROSE minority class probability: $p=0.5$ for SVMs; $p=0.38$ for gradient boosting, naïve Bayes, and linear regression in the primary cohort; $p=0.45$ for gradient boosting, naïve Bayes, and linear regression in the study cohort. We highlighted in italic and with an asterisk * the top result for each statistical indicator. We did not report the results of linear regression trained on the primary cohort and the results of the linear SVM on both the cohorts because these methods predicted all positives in the validation cohort.

#### Train on the primary or study cohort, and test on the external validation cohort

The final part of our analysis involved the attempt to use our trained models to make survival predictions on an external dataset. In a real case scenario, in fact, physicians and medical doctors would apply our approach to the data of a new cohort of patients arriving to the hospital, and these patients of course would not be part of the original cohort where to train the models. To address this scenario, we performed an additional analysis where we trained our models on the Norwegian primary cohort or study cohort of Knoop et al.^95^ and we tested them on the South Korean external validation cohort by Lee et al.^96^. We reported the results in Table 7.

As one can noticed, the algorithms we employed were able to correctly predict most of the survived patients and to make most of correct predctions, obtaining PR AUC scores ranging from 0.821 (radial SVM trained on the primary cohort) to 0.863 (gradient boosting trained on the study cohort). Naïve Bayes obtained the top score for PR AUC when trained on the primary cohort (0.848), while gradient boosting achieved the top PR AUC when trained on the study cohort (0.863). Because of the imbalance of the cohorts, all the methods achieved high scores for positive data instances (sensitivity and precision) but low scores for negative data instances (specificity and NPV). Naïve Bayes achieved the top specificity both when trained on the primary cohort (0.415) and when trained on the study cohort (0.386).

Differently from the other tests we made, some methods failed in correctly predicting any negative data instances: the linear SVM method classified all the validation set data instances as positive, both when trained on the primary cohort and on the study cohort, while the linear regression did the same for primary cohort. This aspect suggests additional future studies in the theoretical machine learning field about the behavior of these algorithms.

These results show, additionally, the level of generalizability of our approach, that is able to correctly predict survived patients just from sex, age, and septic episode even when our models are trained and tested on two different cohorts.

## Discussion

Our results show that machine learning applied to minimal clinical records of patients diagnosed with sepsis, containing only age, sex, and number of septic episode, is sufficient to predict the survival outcome of the patients themselves. Most of our machine learning methods, in fact, were capable to correctly predict most of the survived patients (very high sensitivity rates) with high confidence probability (very high precision values).

To the best of our knowledge, no other study on sepsis has predicted patient survival outcomes with such little and easily obtainable information; age and sex are immediately available for each patient, while sepsis episode number can be easily found in the patient’s history.

Our finding can be consequential to the way that sepsis is managed around the world. If validated, hospitals will be able to quickly and reliably predict a patient’s survival in few seconds, . allowing for quicker action from the doctors, which is crucial for a quick-to-kill illness like sepsis. The finding will be especially useful to hospitals that lack personnel and machinery, like those in rural or developing areas.

Our findings were not identified by the study of the original Norwegian dataset curators, which instead provides an overall general analysis about the correlation between features of the patients’ cohort^87^, and not even by the study of the validation cohort^96^.

As a limitation, we have to report that, even if our machine learning methods resulted being effective in identifying the admissions of survived patients, the same cannot be said for the admissions of deceased patients. Our data mining techniques, in fact, were able to correctly predict most of the admissions of the deceased patients (high TN rates), but with low diagnostic proportions (low NPVs)^98^. We believe this drawback of our study is due to the huge imbalance of the datasets: during training, the machine learning methods do not see enough negative elements, and therefore they generate many false negatives when making predictions on the test set. We tried to tackle this problem with ROSE oversampling^97^, which improved the situation, but did not solve the issue. This drawback is critical because the patients who are more likely to decease are the ones who need urgent therapies and cures in a hospital setting. We hope to overcome this issue in the future by employing other oversampling techniques.

We also have to report that the absence of a temporal feature expressing the time passed between a septic episode and decease has been a limitation for this study. The presence of this time feature, in fact, would have allowed us to make time-related predictions which would have higher impact in a hospital setting, by helping doctors understanding which patients are more in need of immediate help.

In the future, we plan to further investigate the theme of the minimal clinical record for computational prediction of survival on other diseases such as cervical cancer^99^, neuroblastoma^100^, breast cancer^101^, and amyotrophic lateral sclerosis^102^.

## Methods

In this section, we briefly describe the traditional biostatistics tests we employed to detect correlation between each clinical feature and survival target (Biostatistics univariate tests), and the machine learning methods we used to predict survival (Machine learning classifiers).

We implemented our software code with the free open source R programming language and platform^103^, and made it publicly available online on GitHub (Data and software availability).

### Biostatistics univariate tests

To identify preliminary associations between feature (age, sex, septic episode number) and target (survival), we performed univariate biostatistics analyses. We used the Anderson–Darling test^104^ to test for normality of continuous variables. As the normality assumptions were not met, we employed the Mann–Whitney *U* test^105^ to evaluate associations between the continuous features and survival. We used the chi-squared ($\chi ^2$) test^106^ to evaluate the association between sex and survival. We considered *p*-values less than 0.05 as statistically significant.

For both the Mann–Whitney *U* test and the chi-squared test, a low *p*-value (close to 0) means that the two analyzed features strongly relate to each other, while a high *p*-value (close to 1), instead, means there is no correlation^107^.

### Machine learning classifiers

To predict the survival of patients from only three features, we initially employed function approximation methods^108^, trying to frame this scientific problem into a linear setting, with a mathematical formula such as $y = f(x, w, z)$ where where *y* is survival, *x* is age, *w* is sex, and *z* episode number. After several attempts, however, we realized that this problem could not be solved through a simple linear function with three variables, and therefore decided to take advantage of machine learning.

We employed five machine learning classifiers from four different method families: linear regression^109^, support vector machine with linear kernel (linear SVM)^110^, support vector machine with radial kernel (radial SVM)^111^, gradient boosting^112^, and naïve Bayes^113^.

We first chose linear regression because it is a baseline statistical model and one of the simplest methods in computational intelligence; starting an analysis with a simple method is considered a good practice in machine learning^114^. We then chose two support vector machines with different kernels (linear and Gaussian radial), because they can project data into a hyperplane suitable for classification. After that, we tried gradient boosting, an ensemble boosting method capable of training several weak classifiers to build a strong one. Finally, we employed a probabilistic classifier, such as naïve Bayes, which is based on the Bayesian conditional probability and can estimate how likely a data instance can belong to a class.

All these methods have shown their effectiveness in binary classification of biomedical data in the past, and therefore represented suitable candidates for this study as well.

We applied each algorithm 100 times both to the primary cohort and the study cohort and reported the mean result (Results). For methods that needed hyper-parameter optimization (linear SVM and radial SVM), we split the dataset into 60% randomly selected admissions for the training set, 20% randomly selected admissions for the validation set, and 20% remaining admissions for the test set. To choose the top hyper-parameter *C*, we used a grid search and selected the model that generated the highest Matthews correlation coefficient^114,115^. For the other methods (linear regression, naïve Bayes, and gradient boosting), instead, we severed the dataset into 80% randomly selected data instances for the training set, and 20% remaining data instances for the test set.

For each of the 100 executions, our script randomly chose admissions for the training set and for the test set (and for the validation set, in the case of hyper-parameter optimization) from the complete original primary cohort or study cohort. We trained each model on the training set (and validated it on the validation set, in the case of hyper-parameter optimization), and we then applied the model to the test set. Given the different selections of admissions for the dataset splits, each script execution generated slightly different results even when employing the same method.

Because of the huge imbalance of the datasets (92.65% positives and 7.35% negatives in the primary cohort, and 81.07% positives 18.93% negatives in the study cohort), we had to employ an oversampling technique at each execution, to make the training set more balanced. We applied the Randomly Over Sampling Examples (ROSE) method^97^, which creates and adds artificial synthetic data instances of the minority class (the deceased patients, in our datasets) to the training sets. Since we split the datasets into training set, validation set, and test set for the support vector machines, and just into training set and test set for the other methods, we had to select different optimized probability values for the ROSE minority class for these two groups of algorithms.

We measured the classifiers’ performances by using typical confusion matrix evaluation scores such as Matthews correlation coefficient (MCC), receiver operating characteristic area under the curve (ROC AUC), precision recall area under the curve (PR AUC), and other ones. Since our main goal is to correctly predict the survival of patients, we ranked the results based on the PR AUCs, which highlight the true positive rates and positive predictive values reached by each method^116^.

## Supplementary information

Supplementary Information.

## Data Availability

The dataset of the primary cohort and of the study cohort^95^ used in this study is publicly available at: https://plos.figshare.com/articles/Epidemiology_and_impact_on_all-cause_mortality_of_sepsis_in_Norwegian_hospitals_A_national_retrospective_study/5613424. The dataset of the validation cohort^96^ used in this study is publicly available at: https://figshare.com/articles/Severe_persistent_hypocholesterolemia_after_emergency_gastrointestinal_surgery_predicts_in-hospital_mortality_in_critically_ill_patients_with_diffuse_peritonitis/6770660. Our software code is publicly available at: https://github.com/davidechicco/sepsis_survival_from_age_sex_episode
