# Supplementary material for: Survival prediction of patients with sepsis from age, sex, and septic episode number alone
Source: Sci Rep. 2020 Oct 13;10:17156. doi: 10.1038/s41598-020-73558-3 (PMC7555553; doi:10.1038/s41598-020-73558-3)
Supplement: Supplementary file 1 — Supplementary Information. [file 41598_2020_73558_MOESM1_ESM.pdf]

Survival prediction of patients with sepsis from age, sex, and septic episode number alone

Davide Chicco[1,*] and Giuseppe Jurman[2]

[1,*] Krembil Research Institute, Toronto, Ontario, Canada
[2] Fondazione Bruno Kessler, Trento, Italy

* davidechicco@davidechicco.it

Supplementary Information

Supplementary Information

Binary statistical rates

List of statistical rates and their formulas:

$$\text{MCC} = \frac{TP \cdot TN - FP \cdot FN}{\sqrt{(TP+FP) \cdot (TP+FN) \cdot (TN+FP) \cdot (TN+FN)}} \tag{1}$$

(worst value $= -1$; best value $= +1$)

$$\text{F}_1 \text{ score} = \frac{2 \cdot TP}{2 \cdot TP + FP + FN} \tag{2}$$

(worst value $= 0$; best value $= 1$)

$$\text{accuracy} = \frac{TP + TN}{TP + FN + TN + FP} \tag{3}$$

(worst value $= 0$; best value $= 1$)

$$\text{true positive rate (TP rate) = recall = sensitivity} = \frac{TP}{TP + FN} \tag{4}$$

(worst value $= 0$; best value $= 1$)

$$\text{true negative rate (TN rate) = specificity} = \frac{TN}{TN + FP} \tag{5}$$

(worst value $= 0$; best value $= 1$)

$$\text{positive predictive value (PPV) = precision} = \frac{TP}{TP + FP} \tag{6}$$

(worst value $= 0$; best value $= 1$)

$$\text{negative predictive value (NPV)} = \frac{TN}{TN + FN} \tag{7}$$

(worst value $= 0$; best value $= 1$)

$$\text{precision-recall (PR) AUC} = \begin{cases} true\ positive\ rate & on\ the\ x\ axis \\ precision & on\ the\ y\ axis \end{cases} \tag{8}$$

(worst value $= 0$; best value $= 1$)

$$\text{ROC AUC} = \begin{cases} false\ positive\ rate & on\ the\ x\ axis \\ true\ positive\ rate & on\ the\ y\ axis \end{cases} \tag{9}$$

(worst value $= 0$; best value $= 1$)

Survival prediction results with standard deviation

method	value	PR AUC	ROC AUC	TP rate	TN rate	PPV	NPV	MCC	F_1 score	accuracy
train and test on the primary cohort:										
Radial SVM	mean	0.966	0.701	0.492	0.807	0.97	0.112	0.157	0.652	0.515
Radial SVM	σ	0.002	0.006	0.033	0.031	0.003	0.004	0.005	0.029	0.028
Gradient boosting	mean	0.966	0.687	0.611	0.673	0.959	0.121	0.151	0.746	0.615
Gradient boosting	σ	0.001	0.006	0.033	0.041	0.003	0.004	0.007	0.024	0.028
Naive Bayes	mean	0.957	0.641	0.400	0.882	0.977	0.105	0.152	0.568	0.436
Naive Bayes	σ	0.001	0.004	0.005	0.008	0.002	0.002	0.004	0.005	0.004
Linear regression	mean	0.955	0.651	0.532	0.770	0.967	0.116	0.158	0.686	0.549
Linear regression	σ	0.001	0.005	0.005	0.010	0.001	0.003	0.005	0.004	0.004
Linear SVM	mean	0.968	0.706	0.325	0.933	0.984	0.099	0.146	0.489	0.370
Linear SVM	σ	0.001	0.005	0.005	0.006	0.001	0.002	0.004	0.005	0.004
train and test on the study cohort:										
Linear SVM	mean	0.860	0.586	0.205	0.898	0.896	0.210	0.104	0.333	0.337
Linear SVM	σ	0.007	0.010	0.022	0.021	0.013	0.008	0.013	0.028	0.016
Radial SVM	mean	0.858	0.586	0.408	0.718	0.861	0.222	0.102	0.553	0.467
Radial SVM	σ	0.007	0.010	0.031	0.034	0.010	0.009	0.014	0.028	0.020
Gradient boosting	mean	0.856	0.574	0.517	0.595	0.847	0.225	0.090	0.635	0.532
Gradient boosting	σ	0.006	0.011	0.103	0.112	0.015	0.010	0.019	0.083	0.063
Naive Bayes	mean	0.844	0.559	0.294	0.823	0.877	0.214	0.104	0.440	0.394
Naive Bayes	σ	0.006	0.007	0.012	0.017	0.009	0.008	0.013	0.014	0.009
Linear regression	mean	0.841	0.565	0.470	0.661	0.856	0.225	0.103	0.606	0.506
Linear regression	σ	0.007	0.009	0.010	0.017	0.008	0.009	0.014	0.009	0.008

Table S1. Results of the survival prediction made with machine learning classifiers, including standard deviation, with training phase and testing phase done on the Norwegian primary cohort and study cohort. Mean results of 100 executions with random selection of the elements in the training set and test set, with ROSE oversampling applied to the training set. In this analysis, both the training phase and the testing phase happened on the primary cohort or on the study cohort, respectively. σ: standard deviation. Admissions of survived patients: positives data instances (class 1). Admissions of deceased patients: negative data instances (class 0). Linear SVM: support vector machine with linear kernel. Optimized cost regularization hyper-parameter of the linear SVM, most frequently selected C by the MCC-based grid search: $C = 0.01$ for primary cohort (63 times out of 100) and $C = 0.001$ for study cohort (51 times out of 100). Radial SVM: support vector machine with radial Gaussian kernel. Optimized cost regularization of the radial SVM, most frequently selected C by the MCC-based grid search: $C = 0.1$ for the primary cohort (56 times out of 100) and for the study cohort (51 times out of 100). MCC: Matthews correlation coefficient. MCC worst value $= -1$ and best value $= +1$. TP rate: true positive rate, sensitivity, recall. TN rate: true negative rate, specificity. PR: precision-recall curve. PPV: positive predictive value, precision. NPV: negative predictive value. ROC: receiver operating characteristic curve. AUC: area under the curve. F_1 score, accuracy, TP rate, TN rate, PPV, NPV, PR AUC, ROC AUC: worst value $= 0$ and best value $= +1$. ROSE minority class probability: $p = 0.5$ for SVMs; $p = 0.38$ for gradient boosting, naïve Bayes, and linear regression in the primary cohort; $p = 0.45$ for gradient boosting, naïve Bayes, and linear regression in the study cohort. each statistical indicator.

train and test on the validation cohort:										
method	value	PR AUC	ROC AUC	TP rate	TN rate	PPV	NPV	MCC	F_1 score	accuracy
Linear SVM	mean	0.899	0.676	0.911	0.388	0.873	0.490	+0.309	0.889	0.818
Linear SVM	σ	0.071	0.165	0.080	0.265	0.069	0.257	+0.261	0.059	0.084
Naïve Bayes	mean	0.887	0.713	0.899	0.527	0.891	0.538	+0.417	0.893	0.828
Naïve Bayes	σ	0.064	0.116	0.061	0.226	0.064	0.216	+0.200	0.048	0.069
Gradient boosting	mean	0.883	0.682	0.912	0.448	0.885	0.540	+0.378	0.895	0.828
Gradient boosting	σ	0.071	0.126	0.066	0.233	0.063	0.264	+0.219	0.044	0.064
Linear regression	mean	0.880	0.689	0.849	0.530	0.885	0.458	+0.350	0.863	0.788
Linear regression	σ	0.071	0.126	0.101	0.237	0.072	0.213	+0.213	0.071	0.096
Radial SVM	mean	0.873	0.642	0.929	0.226	0.849	0.465	+0.179	0.883	0.806
Radial SVM	σ	0.087	0.176	0.100	0.234	0.070	0.277	+0.234	0.072	0.092

Table S2. Results of the survival prediction made with machine learning classifiers, including standard deviation, on the South Korean external validation cohort. Mean results of 100 executions with random selection of the elements in the training set and test set, with ROSE oversampling applied to the training set. In this analysis, both the training phase and the testing phase happened on the validation cohort. σ: standard deviation. Admissions of survived patients: positives data instances (class 1). Admissions of deceased patients: negative data instances (class 0). Linear SVM: support vector machine with linear kernel. Optimized cost regularization hyper-parameter of the linear SVM, most frequently selected C by the MCC-based grid search: $C = 0.1$ (59 times out of 100). Radial SVM: support vector machine with radial Gaussian kernel. Optimized cost regularization of the radial SVM, most frequently selected C by the MCC-based grid search: $C = 0.1$ (70 times out of 100). MCC: Matthews correlation coefficient. MCC worst value $= -1$ and best value $= +1$. TP rate: true positive rate, sensitivity, recall. TN rate: true negative rate, specificity. PR: precision-recall curve. PPV: positive predictive value, precision. NPV: negative predictive value. ROC: receiver operating characteristic curve. AUC: area under the curve. F_1 score, accuracy, TP rate, TN rate, PPV, NPV, PR AUC, ROC AUC: worst value $= 0$ and best value $= +1$. ROSE p-value: 0.5 for all.

method	value	PR AUC	ROC AUC	TP rate	TN rate	PPV	NPV	MCC	F_1 score	accuracy
train on primary cohort and test on validation cohort:										
Naïve Bayes	mean	0.848	0.565	0.715	0.415	0.852	0.236	+0.107	0.777	0.663
Naïve Bayes	σ	0.005	0.013	0.020	0.030	0.006	0.013	+0.022	0.012	0.014
Gradient boosting	mean	0.843	0.527	0.953	0.035	0.823	0.123	–0.018	0.882	0.792
Gradient boosting	σ	0.009	0.018	0.060	0.058	0.006	0.077	+0.044	0.028	0.042
Radial SVM	mean	0.821	0.514	0.949	0.013	0.819	0.040	–0.068	0.879	0.785
Radial SVM	σ	0.019	0.036	0.032	0.031	0.006	0.093	+0.058	0.016	0.026
0.000	–0.087	0.883	0.790							
	0.000	+0.009	0.004	0.007						
train on study cohort and test on validation cohort:										
Gradient boosting	mean	0.863	0.552	0.973	0.061	0.830	0.739	+0.130	0.895	0.814
Gradient boosting	σ	0.017	0.029	0.045	0.038	0.007	0.392	+0.124	0.022	0.035
Naïve Bayes	mean	0.848	0.566	0.747	0.386	0.851	0.244	+0.113	0.795	0.683
Naïve Bayes	σ	0.002	0.007	0.007	0.019	0.003	0.005	+0.011	0.004	0.004
Radial SVM	mean	0.829	0.537	0.955	0.011	0.820	0.043	–0.068	0.882	0.789
Radial SVM	σ	0.007	0.012	0.008	0.023	0.004	0.087	+0.047	0.005	0.008
Linear regression	mean	0.824	0.499	0.956	0.042	0.824	0.166	–0.005	0.885	0.796
Linear regression	σ	0.000	0.000	0.001	0.000	0.000	0.002	+0.001	0.000	0.001

Table S3. Results of the survival prediction made with machine learning classifiers, including standard deviation, with training phase done on the Norwegian primary cohort or study cohort and testing phase done on the South Korean external validation cohort. Mean results of 100 executions with random selection of the elements in the training set and test set, with ROSE oversampling applied to the training set. σ: standard deviation. Admissions of survived patients: positives data instances (class 1). Admissions of deceased patients: negative data instances (class 0). Linear SVM: support vector machine with linear kernel. Optimized cost regularization hyper-parameter of the linear SVM, most frequently selected C by the MCC-based grid search: $C = 0.01$ for primary cohort (63 times out of 100) and $C = 0.001$ for study cohort (51 times out of 100). Radial SVM: support vector machine with radial Gaussian kernel. Optimized cost regularization of the radial SVM, most frequently selected C by the MCC-based grid search: $C = 0.1$ for the primary cohort (56 times out of 100) and for the study cohort (51 times out of 100). MCC: Matthews correlation coefficient. MCC worst value $= -1$ and best value $= +1$. TP rate: true positive rate, sensitivity, recall. TN rate: true negative rate, specificity. PR: precision-recall curve. PPV: positive predictive value, precision. NPV: negative predictive value. ROC: receiver operating characteristic curve. AUC: area under the curve. F_1 score, accuracy, TP rate, TN rate, PPV, NPV, PR AUC, ROC AUC: worst value $= 0$ and best value $= +1$. ROSE minority class probability: $p = 0.5$ for SVMs; $p = 0.38$ for gradient boosting, naïve Bayes, and linear regression in the primary cohort; $p = 0.45$ for gradient boosting, naïve Bayes, and linear regression in the study cohort. We did not report the results of linear regression trained on the primary cohort and the results of the linear SVM on both the cohorts because these methods predicted all positives in the validation cohort. each statistical indicator.
